# Draft Genome Sequence of Enterobacter sp. Strain OLF, a Colonizer of Olive Flies

**DOI:** 10.1128/MRA.01068-18

**Published:** 2018-09-06

**Authors:** Anne M. Estes, David J. Hearn, Suvarna Nadendla, Elizabeth A. Pierson, Julie C. Dunning Hotopp

**Affiliations:** a Institute for Genome Sciences, University of Maryland School of Medicine, Baltimore, Maryland, USA; b Department of Biological Sciences, Towson University, Towson, Maryland, USA; c Department of Horticultural Sciences, Texas A&M University, College Station, Texas, USA; d Department of Microbiology and Immunology, University of Maryland School of Medicine, Baltimore, Maryland, USA; University of Delaware

## Abstract

Enterobacter sp. strain OLF colonizes laboratory-reared and wild individuals of the olive fruit fly Bactrocera oleae. The 5.07-kbp genome sequence of Enterobacter sp. strain OLF encodes metabolic pathways that allow the bacterium to partially supplement the diet of the olive fly when its dominant endosymbiont, Erwinia dacicola, is absent.

## Abstract (precis)

Enterobacter sp. strain OLF colonizes laboratory-reared and wild individuals of the olive fruit fly Bactrocera oleae.

## ANNOUNCEMENT

Enterobacter sp. strain OLF was sequenced due to its prevalence in laboratory-reared and, occasionally, wild olive fly populations (1). It is the only culturable isolate obtained from olive fly abdomen homogenate (2). We are interested in its potential as a probiotic replacement or supplement for olive flies in mass rearing activities aimed at pest control that are lacking Erwinia dacicola, the dominant beneficial endosymbiont (3).

Enterobacter sp. strain OLF was originally isolated from the abdomen of a wild male olive fly from the western United States (2). Only round, smooth, white colonies of Enterobacter sp. strain OLF were found on LB plates after 24 h at 27°C. Enterobacter sp. strain OLF can grow between 27 and 37°C (2). Based on a Bayesian analysis (2) of sequences concatenated from the 16S rRNA (∼1,400 bp), *ompA* (∼900 bp), and *recA* (∼870 bp), it was determined to be in the family Gammaproteobacteria and, more specifically, the genus Enterobacter.

To isolate DNA for genome sequencing, a single colony of Enterobacter sp. strain OLF was grown overnight in LB broth with aeration at 37°C. Approximately 8 µg of DNA was purified using the Gram-negative protocol in the DNeasy kit (Qiagen, Valencia, CA), which was used by the Arizona Genomics Institute at the University of Arizona to construct 75-bp paired-end Illumina libraries. Libraries were tagged and multiplexed for sequencing on one lane of an Illumina Genome Analyzer II. The sequencing run generated 28,937,299 paired-end reads. Except where otherwise specified, default parameters were used for all software. Sequences were assembled into contigs using *de novo* assembly in ABySS-pe (version 1.2.5) with 4 different k-mer sizes (40, 45, 50, and 55) with a cutoff of 300 bp. The k50 assembly was selected based on closest similarity to Enterobacter cloacae subsp. cloacae ATCC 13047, the closest sequenced relative, and yielded 67 contigs with an *N*_50_ value of 180,401 bp, GC content of 55.1%, a >300× sequencing depth, and a maximum contig size of 362 kbp. The Enterobacter sp. strain OLF genome was annotated using the IGS Annotation Engine (4) with Glimmer version 3.02 (5), yielding 4,760 coding sequences, of which 89.1% encoded proteins. The genome encodes the ability to supplement amino acids and vitamins missing from the olive fruit on which the larvae feed, supporting further testing of Enterobacter sp. strain OLF as a probiotic.

### Data availability.

The reads, assembly, and annotation can be accessed through BioProject number PRJNA288712 and BioSample number SAMN03836970. This whole-genome shotgun project has been deposited at DDBJ/ENA/GenBank under the accession number LJAN00000000. The version described in this paper is the first version, LJAN01000000.
